# Supplementary material for: Transgenerational effects enhance specific immune response in a wild passerine
Source: PeerJ. 2016 Mar 31;4:e1766. doi: 10.7717/peerj.1766 (PMC4824879; doi:10.7717/peerj.1766)

TRANSGENERATIONAL EFFECTS ENHANCE SPECIFIC IMMUNE RESPONSE IN A WILD PASSERINE

Juli Broggi^1,2,4^, Ramón C. Soriguer^1,3^ and Jordi Figuerola^1,3^

1. Estación Biológica Doñana, CSIC. Avda. Americo Vespucio s/n. 41092 Sevilla, Spain.

2. Research Unit of Biodiversity (UO, CSIC, PA), Universidad de Oviedo, 33600 Mieres, Spain.

3. CIBER Epidemiología y Salud Pública (CIBERESP), Spain

**4**. **Author for correspondence**. Department of Biology, Section of Evolutionary Ecology, University of Lund, S-223 62 Lund, Sweden. Email: [julibroggi@gmail.com](mailto:julibroggi@gmail.com)

Table 1.

Results from the general linear mixed model on Newcastle disease virus (NDV) antibody concentration of house sparrow chicks in relation to main effects, primary covariates and their interactions. Predictors from each model are shown, together with the corresponding F values, DF and P values. Significant predictors are presented in bold, and AIC values are presented for each model. Parameter estimates ± S.E are presented for significant predictors, and for categorical predictors values for control treatment are presented for zero-adjusted experimental treatment. TR denotes treatment, S for sampling date and [NDV-Ab] NDV antibody concentration.

|  | **AIC** | **Predictors** | **F value** | **DF** | **P** | **Estimate±S.E.** |
| --- | --- | --- | --- | --- | --- | --- |
| **Initial** | 358.59 | Mother TR | 0.06 | 1,20 | 0.066 |  |
|  |  | Chick TR | 3.80 | 1,21 | 0.810 |  |
|  | 356.77 | Mother TR | 0.07 | 1,21 | 0.788 |  |
|  |  | Chick TR | 3.83 | 1,19 | 0.065 |  |
|  |  | Mother TR x Chick TR | 0.52 | 1,19 | 0.481 |  |
|  | 315.75 | Mother TR | 0.98 | 1,18 | 0.335 |  |
|  |  | **Chick TR** | **7.39** | **1,18** | **0.014** | **-1.038±0.382** |
|  |  | Mother [NDV-Ab] | 1.06 | 1,18 | 0.317 |  |
|  | 311.87 | Mother TR | 0.83 | 1,18 | 0.373 |  |
|  |  | Chick TR | 0.51 | 1,18 | 0.485 |  |
|  |  | Mother [NDV-Ab] | 0.84 | 1,18 | 0.372 |  |
|  |  | **Chick TR x Mother [NDV-Ab]** | **6.37** | **1,53** | **0.015** | **-0.336±0.133** |
| **Final** | **307.82** | Chick TR | 0.79 | 1,18 | 0.386 |  |
|  |  | Mother TR | 0.06 | 1,17 | 0.810 |  |
|  |  | Mother [NDV-Ab] | 0.19 | 1,17 | 0.666 |  |
|  |  | **Days Chick S-Mother S** | **7.20** | **1,17** | **0.016** | **0.324±0.121** |
|  |  | **Chick TR x Mother [NDV-Ab]** | **6.61** | **1,53** | **0.013** | **-0.341±0.133** |

Table 2.

Results from the general linear mixed models on Newcastle disease virus (NDV) antibody concentration of house sparrow chicks in relation to several secondary covariates. Predictors from each model are shown, together with the corresponding F values, DF and P values. Significant predictors are presented in bold, and AIC values are presented for each model. Parameter estimates ± S.E are presented for significant predictors, and for categorical predictors values for control treatment are presented for zero-adjusted experimental treatment. TR denotes treatment, S for sampling date and [NDV-Ab] NDV antibody concentration.

|  | **AIC** | **Predictors** | **F value** | **DF** | **P** | **Estimate±S.E.** |
| --- | --- | --- | --- | --- | --- | --- |
|  | 310.11 | Sex | 0.33 | 1,15 | 0.574 |  |
|  | 311.22 | Tarsus (mm) | 2.02 | 1,52 | 0.162 |  |
|  | 311.45 | Body mass (g) | 0.07 | 1,52 | 0.788 |  |
|  | 315.32 | Hatching date | 0.03 | 1,17 | 0.863 |  |
|  | 309.73 | Clutch size | 0.45 | 1,17 | 0.510 |  |
|  | 298.42 | Uric acid (mg/dL) | 0.94 | 1,51 | 0.337 |  |
|  | 295.14 | Chick TR x UAC | 0.03 | 1,50 | 0.872 |  |
|  | 295.11 | Total protein (mg/dL) | 3.23 | 1,51 | 0.078 |  |
|  | 288.66 | Chick TR x TPR | 2.07 | 1,50 | 0.157 |  |
|  | **255.47** | **Carotenoids (mg/L)** | **8.11** | **1,39** | **0.007** | **-1.346±0.473** |
|  | 252.44 | Chick TR x CAR | 1.30 | 1,38 | 0.262 |  |
|  | **288.89** | **TAC (µmol/L)** | **14.68** | **1,51** | **<0.001** | **-1.472±0.384** |
|  | **281.95** | **Chick TR x TAC** | **5.95** | **1,50** | **0.018** | **1.734±0.711** |

Table 3.

Results from the general linear mixed models on PHA inflammatory test of house sparrow chicks in relation to main effects, primary covariates and their interactions. Predictors included to the final model, together with the corresponding F values, DF and P values, and parameter estimates ± S.E when not categorical. Significant predictors are presented in bold, and AIC values are presented for each model. TR denotes treatment, S for sampling date and [NDV-Ab] NDV antibody concentration. Time PHA denotes time between first and second PHA measurements.

|  | **AIC** | **Predictors** | **F value** | **DF** | **P** | **Estimate±S.E.** |
| --- | --- | --- | --- | --- | --- | --- |
| **Initial** | 43.45 | Mother TR | 0.01 | 1,9 | 0.926 |  |
|  |  | Chick TR | 0.02 | 1,21 | 0.878 |  |
|  | 44.57 | Mother TR | 0.01 | 1,9 | 0.923 |  |
|  |  | Chick TR | 0.00 | 1,20 | 0.982 |  |
|  |  | Mother TR x Chick TR | 0.28 | 1,20 | 0.604 |  |
|  | 46.87 | Mother TR | 0.12 | 1,8 | 0.741 |  |
|  |  | Chick TR | 0.01 | 1,21 | 0.907 |  |
|  |  | Days Chick S-Mother S | 0.94 | 1,8 | 0.361 |  |
|  | 47.91 | Mother TR | 0.14 | 1,8 | 0.719 |  |
|  |  | Chick TR | 0.03 | 1,10 | 0.867 |  |
|  |  | Time PHA | 1.41 | 1,8 | 0.270 |  |

Table 4.

Results from the general linear mixed models on PHA inflammatory test of house sparrow chicks in relation to several secondary covariates. Predictors included in the final model as covariates, together with the corresponding F values, DF and P values, and parameter estimates ± S.E for continuous variables. Significant predictors are presented in bold, and AIC values are presented for each model. TR denotes treatment, S for sampling date and [NDV-Ab] NDV antibody concentration.

|  | **AIC** | **Predictors** | **F value** | **DF** | **P** | **Estimate±S.E.** |
| --- | --- | --- | --- | --- | --- | --- |
|  | 45.99 | Sex | 0.04 | 1,18 | 0.843 |  |
|  | 46.02 | Tarsus (mm) | 1.13 | 1,29 | 0.296 |  |
|  | **44.54** | **Body mass (g)** | **5.05** | **1,29** | **0.032** | **-0.055±0.024** |
|  | 49.02 | Chick TR x Body mass | 0.35 | 1,28 | 0.560 |  |
|  | 49.41 | Hatching date | 0.32 | 1,8 | 0.588 |  |
|  | 43.27 | Clutch size | 2.04 | 1,8 | 0.192 |  |
|  | 37.17 | Uric acid (mg/dL) | 0.48 | 1,28 | 0.495 |  |
|  | 35.31 | Total protein (mg/dL) | 0.77 | 1,28 | 0.388 |  |
|  | 28.62 | Carotenoids (mg/L) | 3.13 | 1,20 | 0.092 |  |
|  | 37.06 | TAC (µmol/L) | 2.06 | 1,28 | 0.162 |  |

Table 5.

Results from the general linear mixed models on body mass increase between challenge and sampling of house sparrow chicks in relation to main effects, primary covariates and their interactions. Predictors are presented with the corresponding F values, DF and P values, and parameter estimates ± S.E when continuous. Significant predictors are presented in bold, and AIC values are presented for each model. TR denotes treatment, S for sampling date and [NDV-Ab] NDV antibody concentration. Time denotes days between first and second body mass measurements.

|  | **AIC** | **Predictors** | **F value** | **DF** | **P** | **Estimate±S.E.** |
| --- | --- | --- | --- | --- | --- | --- |
| **Initial** | 408.50 | Mother TR | 0.80 | 1,21 | 0.382 |  |
|  |  | Chick TR | 0.71 | 1,21 | 0.410 |  |
|  | 407.13 | Mother TR | 0.80 | 1,21 | 0.380 |  |
|  |  | Chick TR | 0.68 | 1,20 | 0.418 |  |
|  |  | Mother TR x Chick TR | 0.02 | 1,20 | 0.889 |  |
|  | 360.30 | Mother TR | 2.50 | 1,18 | 0.132 |  |
|  |  | Chick TR | 1.34 | 1,19 | 0.262 |  |
|  |  | Mother [NDV-Ab] | 4.16 | 1,18 | 0.056 |  |
| **Final** | **352.60** | Mother TR | 0.34 | 1,17 | 0.567 |  |
|  |  | Chick TR | 1.18 | 1,19 | 0.291 |  |
|  |  | Mother [NDV-Ab] | 1.16 | 1,17 | 0.297 |  |
|  |  | **Time** | **9.77** | **1,17** | **0.006** | **0.977±0.313** |

Table 6.

Results from the general linear mixed models on body mass increase between challenge and sampling of house sparrow chicks in relation to several secondary covariates. Predictors included in the final model as covariates, together with the corresponding F values, DF and P values, and parameter estimates ± S.E for continuous variables. Significant predictors are presented in bold, and AIC values are presented for each model. TR denotes treatment.

|  | **AIC** | **Predictors** | **F value** | **DF** | **P** | **Estimate±S.E.** |
| --- | --- | --- | --- | --- | --- | --- |
|  | 395.55 | Sex | 0.62 | 1,17 | 0.441 |  |
|  | **392.26** | **Tarsus (mm)** | **4.96** | **1,63** | **0.030** | **0.597±0.268** |
|  | 391.72 | Chick TR x Tarsus | 0.66 | 1,62 | 0.420 |  |
|  | 399.72 | Hatching date | 0.35 | 1,19 | 0.561 |  |
|  | 395.02 | Clutch size | 0.00 | 1,19 | 0.985 |  |
|  | 363.80 | Uric acid (mg/dL) | 0.01 | 1,60 | 0.933 |  |
|  | 362.16 | Total protein (mg/dL) | 0.16 | 1,60 | 0.687 |  |
|  | 308.03 | Carotenoids (mg/L) | 0.06 | 1,46 | 0.806 |  |
|  | 365.37 | TAC (µmol/L) | 0.46 | 1,60 | 0.498 |  |

Table 7.

Results from the general linear mixed models on house sparrow chick survival until fledging of in relation to main effects, primary covariates and their interactions, and secondary covariates. Predictors are presented for each model, together with the corresponding F values, DF and P values, and parameter estimates ± S.E when not categorical. Significant predictors are presented in bold, and generalized Chi^2^ values are presented for each model. TR denotes treatment and [NDV-Ab] NDV antibody concentration.

|  | **Gen. Chi^2^** | **Predictors** | **F value** | **DF** | **P** | **Estimate±S.E.** |
| --- | --- | --- | --- | --- | --- | --- |
| **Initial** | 91.46 | Mother TR | 1.03 | 1,86 | 0.313 |  |
|  |  | Chick TR | 0.02 | 1,86 | 0.897 |  |
|  | 91.0 | Mother TR | 0.94 | 1,85 | 0.335 |  |
|  |  | Chick TR | 0.02 | 1,85 | 0.893 |  |
|  |  | Mother TR x Chick TR | 0.33 | 1,85 | 0.567 |  |
|  | 78.23 | Mother TR | 1.51 | 1,77 | 0.223 |  |
|  |  | Chick TR | 0.00 | 1,77 | 0.944 |  |
|  |  | Mother [NDV-Ab] | 0.03 | 1,77 | 0.862 |  |
| **Final** | **63.51** | Mother TR | 1.99 | 1,85 | 0.162 |  |
|  |  | Chick TR | 0.21 | 1,85 | 0.649 |  |
|  |  | **Body mass (g)** | **20.34** | **1,85** | **<0.001** | **0.518±0.115** |
|  | 63.27 | Chick TR x Body mass | 0.37 | 1,84 | 0.545 |  |
|  | 62.05 | Clutch size | 2.99 | 1,85 | 0.088 |  |
|  | **70.34** | **Hatching date** | **5.50** | **1,85** | **0.021** | **0.097±0.042** |

Figure 2. Carotenoid concentration in blood (CAR) (2A), and blood total antioxidant capacity (TAC) (2B) in relation to Newcastle disease virus (NDV) antibody titres of house sparrow (Passer domesticus) chicks for the different treatments. Antibody titres are expressed as the log of the inverse of the dilution factor.

2A


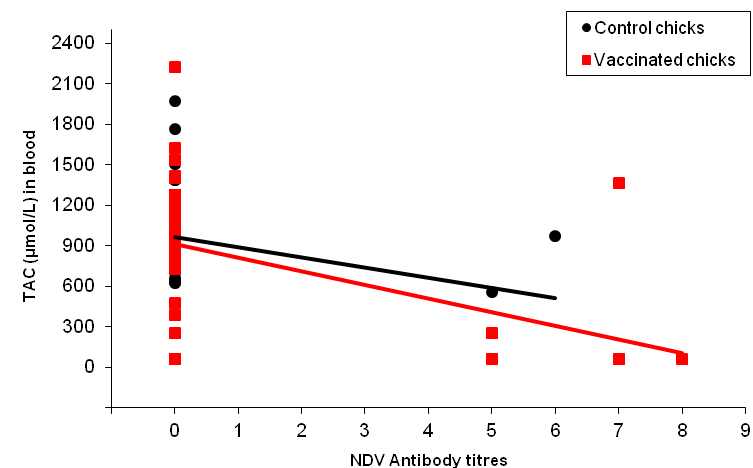


2B


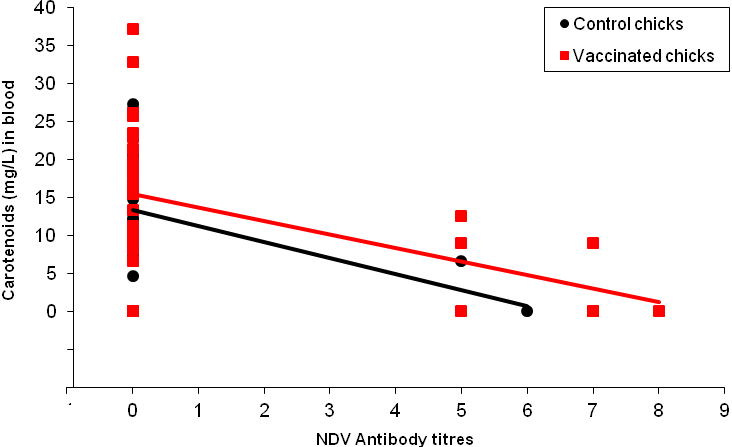

Supplement: Supplemental Information 1 — Details on the non-significant parameter estimates when removed from the model, and figures on the relationship between blood carotenoids and TAC in chicks for the different experimental treatments. [file peerj-04-1766-s001.docx]
